# Prevalence of Vitamin D Deficiency and Its Associations with Skin Color in Pregnant Women in the First Trimester in a Sample from Switzerland

**DOI:** 10.3390/nu9030260

**Published:** 2017-03-10

**Authors:** Aline Richard, Sabine Rohrmann, Katharina C. Quack Lötscher

**Affiliations:** 1 Epidemiology, Biostatistics and Prevention Institute, University of Zurich, Hirschengraben 84, CH-8001 Zurich, Switzerland; richard@patientensicherheit.ch (A.R.); sabine.rohrmann@uzh.ch (S.R.); 2 Clinic of Obstetrics, University Hospital Zurich, Frauenklinikstrasse 10, CH-8091 Zurich, Switzerland

**Keywords:** vitamin D, pregnancy, skin color, vitamin D deficiency, Switzerland

## Abstract

Vitamin D deficiency in pregnancy has negative clinical consequences, such as associations with glucose intolerance, and has been shown to be distributed differently in certain ethnic groups. In some countries, a difference in the rate of vitamin D deficiency was detected in pregnant women depending on their skin color. We examined the prevalence of vitamin D deficiency (<20 ng/mL) in women in early pregnancy in Switzerland and evaluated the association of skin color with vitamin D deficiency. In a single-center cohort study, the validated Fitzpatrick scale and objective melanin index were used to determine skin color. Of the 204 pregnant women included, 63% were vitamin D deficient. The mean serum 25-hydroxyvitamin D concentration was 26.1 ng/mL (95% confidence interval (CI) 24.8–27.4) in vitamin D–sufficient women and 10.5 ng/mL (95% CI 9.7–11.5) in women with deficiency. In the most parsimonious model, women with dark skin color were statistically significantly more often vitamin D deficient compared to women with light skin color (OR 2.60; 95% CI 1.08–6.22; adjusted for age, season, vitamin D supplement use, body mass index, smoking, parity). This calls for more intense counseling as one policy option to improve vitamin D status during pregnancy, i.e., use of vitamin D supplements during pregnancy, in particular for women with darker skin color.

## 1. Introduction

During the last century, vitamin D fortification programs have largely eradicated the health risks of vitamin D deficiency such as rickets and osteomalacia from western populations. However, vitamin D deficiency (<20 ng/mL) is reemerging and suboptimal vitamin D blood levels are widespread in industrialized nations, specifically in women with darker skin color [1,2,3,4,5,6,7]. A suboptimal vitamin D level is thought to be associated with a range of diseases such as cardiovascular disease and diabetes [8] as well as with the risk of several types of cancer or depression [9,10]. Cholecalciferol (vitamin D3) is synthesized in the skin by sunlight (UVB) from 7-dehydrocholesterol, followed by transformation to the active form 25-hydroxyvitamin D ((25(OH)D) in the liver. In a further step, 25(OH)D is metabolized into the physiologically active 1,25-dihydroxyvitamin D (1,25(OH)2D) in the kidney. As 25(OH)D has a half-life of 15 days, which is longer than that of 1,25(OH)2D, it is considered to be the better indicator of vitamin D status. Individuals living in countries with less sun exposure might be at higher risk for vitamin D deficiency [11]. Additionally, during winter and spring, sun exposure is low in northern countries. Besides geographic and weather circumstances, several studies showed that personal characteristics affect vitamin D synthesis. Circulating vitamin D concentrations differ by skin color: Individuals with darker skin produce less vitamin D with the same amount of sunlight exposure than individuals with lighter skin color [10,12]. In Europe, estimated vitamin D levels showed a large variation due to risk factors such as immigration from countries with higher sun exposure, low consumption of foods rich in vitamin D or low vitamin D supplementation [13].

In pregnancy, increased calcium and adequate vitamin D levels are required and, thus, pregnant women are, in general, at higher risk of vitamin D deficiency [6]. Vitamin D deficiency in pregnancy has been shown to be associated with a variety of clinical consequences [14,15,16,17] that range from a negative influence on glucose tolerance to an association with preeclampsia. Vitamin D supplementation can improve birth weight in certain ethnic groups [18].

Currently, the vitamin D status of pregnant women living in Switzerland is unknown [19] and, hence, the aim of our study was to evaluate vitamin D levels in pregnant women and to determine the prevalence of vitamin D deficiency. Furthermore, we aimed to address the question of whether the prevalence of vitamin D deficiency differs between women with light or dark skin color, i.e., between specific subgroups of the population living in Switzerland.

## 2. Materials and Methods

### 2.1. Study Population

Between September 2014 and December 2015, 80% of the women visiting the Clinic of Obstetric at the University Hospital Zurich for their first pregnancy visit in the first trimester were recruited for participation in this vitamin D study. The study was approved by the ethics committee of the canton of Zurich, Switzerland (KEK-ZH-Nr. 2013-0213). Exclusion criteria were twin pregnancy, HIV, history of parathyroid, renal or liver disease, chronic malabsorption syndromes or granuloma-forming disorders, age below 18 years or known or suspected drug or alcohol abuse, because they may alter vitamin D metabolism. We collected data of 205 women. Due to one missing information on vitamin D status, our final sample consisted of 204 women.

### 2.2. Vitamin D Blood Samples

After giving informed consent, a blood sample of 10 mL was collected during the routine blood collection of the pregnancy examination. Blood samples were centrifuged and serum was extracted in the Institute of Clinical Chemistry at the University Hospital Zurich within hours after blood sampling. Total 25-hydroxyvitamin D was analyzed on the same day using the vitamin D total-analysis Roche Cobas^®^ electrochemiluminescence immunoassay (Roche Diagnostics, Basel, Switzerland). The method has a detection range of 3.0–70.0 ng/mL for 25(OH) vitamin D and a variation coefficient of 2.2%–6.8%.

Vitamin D deficiency was defined as 25(OH)D concentrations <20 ng/mL vs. sufficiency as ≥20 ng/mL as recommended by the Endocrine Society [20]. The conversion factor to the SI units (nmol/L) is 2.496.

### 2.3. Skin Color

The physician together with the participant filled out a questionnaire. The skin color of the women was assessed according to the classification by Fitzpatrick [21]. This scale allows for differentiating between skin phototypes based on skin reaction to sun exposure. The origin scale consists of six skin types (I to VI). We used an adapted scale, which converged type V (dark brown) and VI (black) into type V due to small numbers in these groups. The classification of skin type was assessed first by showing the participant a picture of the different skin color types (I–IV) and second, by asking on what happens to the untanned skin if it is exposed in the early summer at noon for 45 to 60 min to the sun. Answers varied from (I) “painful sunburn after 24 h and not tanned after one week”; (II) “painful sunburn after 24 h and minimally tanned after one week”; (III) “minimal sunburn after 24 h and uniformly tanned after one week”; (IV) “no sunburn after 24 h and tanned after one week” to (V) “Skin is deeply pigmented brown/black, no sunburn and tanned after one week”. Based on the pictures and questions the women estimated their own skin phototype. Additionally, the interviewer evaluated the skin type. When the classification of pregnant woman and the interviewer disagreed, the rounded arithmetic mean of was used to determine the skin color type. Skin color type was dichotomized into Fitzpatrick scale I to III vs. IV and V.

Furthermore, the skin type was measured with a DSM II ColorMeter (Cortex Technology, Hadsund, Denmark) resulting in a melanin index [22]. The device is a narrow band spectroscopy instrument with a green diode centered on 568 nm and red diode centered on 655 nm. The device was calibrated every week with white balance. Melanin index was measured 3 times on the inner underarm and the arithmetic mean was calculated to categorize melanin in quartiles. Spearman correlation coefficient between melanin index and the Fitzpatrick index was 0.65.

### 2.4. Covariates

Based on the World Bank Map, country of a woman’s origin (place of birth) was grouped into five categories; (1) Switzerland and Germany; (2) Northern America, Northern Europe, Caucasus, Central Asia and New Zealand; (3) Southern Europe, Australia, Latin America and the Caribbean; (4) South- and East Asia and Pacific; and (5) Africa and Middle East. For further analyses these countries were dichotomized into groups 1 and 2 vs. groups 3–5. Further covariates were age, week of pregnancy, parity, gravidity, body mass index (BMI) before pregnancy, actual BMI, educational level of the pregnant woman and her partner (less than compulsory education vs. low (compulsory education) vs. middle (secondary education) vs. high (tertiary education)), smoking status (never vs. former vs. current), season of blood collection (winter vs. spring vs. summer vs. fall), number of days per week spent at least 1 hour outdoors in the past half year, sun protection (never vs. sometimes vs. always), fish consumption (only salmon, tuna, mackerels and herring; at least once per week vs. less), vitamin D supplements intake as recommended 500 IE daily (yes vs. no).

In addition, maternal age, parity, week of pregnancy, body mass index (BMI) before pregnancy and weight gain till the first visit were collected from medical records.

### 2.5. Statistical Analyses

All statistical analyses were conducted using STATA software version 13.1 (College Station, TX, USA). Geometric means and corresponding 95% confidence intervals (CI) were used to illustrate the differences in vitamin D concentrations between light- and dark-skinned individuals. 

Logistic regression analyses were used to determine associations of skin color with vitamin D deficiency. The Akaike Information Criteria (AIC) was used for selecting the final model solution for multivariable adjustment. As a result of the AIC and of dropping variables because of collinearity, we presented the 4 most parsimonious models; (1) adjusted for age; (2) adjusted for age and season, (3) adjusted for age, season, vitamin D supplement intake, BMI and smoking status; and (4) adjusted for age, season, vitamin D supplement intake, BMI, smoking status and parity. Sensitivity-analyses were performed using the dichotomized countries of origin and the dichotomized melanin index (by median) instead of the Fitzpatrick scale. Differences between groups were examined using Anova and *t*-test (*p* < 0.05, two-sided).

## 3. Results

Descriptive characteristics of the 204 women are provided in Table 1 for women with and without vitamin D deficiency. A description by skin type can be found in Supplementary Table S1.

Almost two-thirds of the women were vitamin D deficient. The mean serum vitamin D concentration was 26.1 ng/mL in vitamin D–sufficient women and 10.5 ng/mL in women with deficiency. Light skin color was reported by 88% of the women with sufficient vitamin D levels and by 66.6% with vitamin D deficiency. 

The mean age at blood collection was 31.1 and 29.4 years in vitamin D–sufficient and –deficient women, respectively. About one-third of the women with sufficient vitamin D levels and 14% of vitamin D–deficient women were of German or Swiss origin. Half of the women with a sufficient vitamin D concentration sometimes used sun protection, 13% never used it and 36% always used sun protection. In women with vitamin D deficiency, sun protection was used “never”, “sometimes” or “always” by one-third of women each. Fish consumption was reported by 51% of the women without and by 45% of the women with vitamin D deficiency, and vitamin D supplements intake was reported by 9% of the women without and with vitamin D deficiency, respectively. 

The associations of dark skin color with vitamin D deficiency were assessed by logistic regression using different adjustment models (Table 2).

The AIC fit best for the age-adjusted model with an OR of 3.25 (95% CI 1.46–7.24) for the associations of skin color with vitamin D deficiency. The next model with a good AIC fit included age and season (OR 3.29; 95% CI 1.4–7.36). In the multivariable adjusted model including age, season, vitamin D supplement intake, BMI, smoking status and parity, the OR was 2.60 (95% CI 1.08–6.22). In the sensitivity analysis using dichotomized countries of origin instead of the Fitzpatrick scale, the best AIC fit and the results remained similar (Supplementary Table S2). However, in the sensitivity analysis with the dichotomized melanin index as a proxy for skin color, the AIC fit best for the same model as in our main analysis, but the associations of dark skin color with vitamin D deficiency were attenuated and the results for models 3 and 4 did not remain statistically significant (Supplementary Table S3).

**Figure 1:**
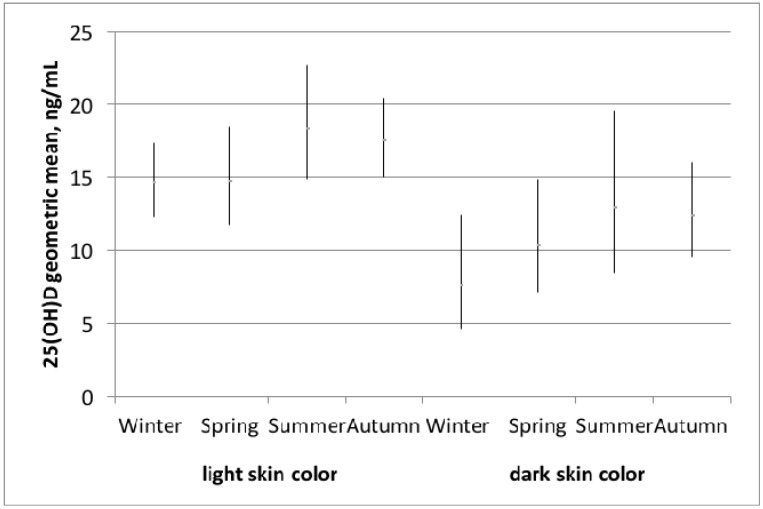
Vitamin D levels stratified by light and dark skin color and season according to the Fitzpatrick scale (I, II, III vs. IV, V) and season (winter = December–February, spring = March–May, summer = June–August, autumn = September–November).

According to the results of the AIC, age and season explained the model best. Figure 1 depicts the geometric mean vitamin D levels by light and dark skin color type (according to the Fitzpatrick scale) stratified by season.

Women with light skin color had the highest vitamin D levels in summer and the lowest in winter (18.4 ng/mL (95% CI 15.0–22.7) and 14.6 ng/mL (95% CI 12.3–17.4), respectively). For women with dark skin color, these levels in summer and winter were lower (12.9 ng/mL (95% CI 8.5–19.6) and 7.6 ng/mL (95% CI 4.6–12.5), respectively). However, differences between seasons were not statistically significant either in light-skinned or in dark-skinned women. 

**Figure 2:**
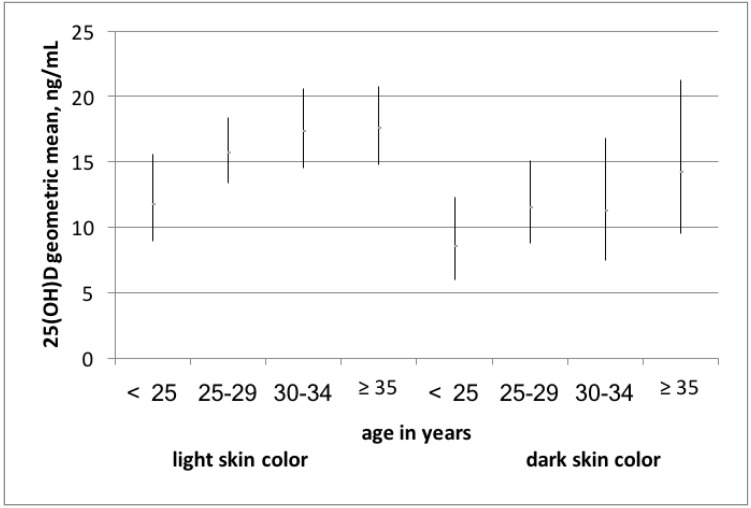
Vitamin D levels stratified by light and dark skin color according to the Fitzpatrick scale and the four age groups.

Younger women had lower vitamin D levels compared to older women with light and with dark skin color (Figure 2).

Vitamin D levels in women younger than 25 years were 11.8 ng/mL (95% CI 8.9–15.6) and 8.6 ng/mL (95% CI 6.0–12.3) in light and dark skin color, respectively. Women aged 35 or above had a mean vitamin D level of 17.6 ng/mL (95% CI 14.8–20.8) (light skin color) and 14.2 ng/mL (95% CI 9.5–21.9) (dark skin color). In dark-skinned women, differences were not statistically significant; in light-skinned women, the *p*-value was 0.06 (Anova).

## 4. Discussion

In our study, almost two-thirds of the pregnant women were vitamin D deficient and dark skin color was associated with a higher prevalence of vitamin D deficiency. This prevalence is almost twice as high as in the normal population (38%) [23].

To our knowledge, the prevalence of vitamin D deficiency in women in the first trimester of pregnancy has not yet been evaluated in Switzerland. A recent systematic review looking at vitamin D deficiency in pregnant women in the Mediterranean region observed a prevalence of vitamin D deficiency (defined as ≤20 ng/mL) in pregnant women ranging from 22.7% to 90.3% [24]. Only four out of 15 studies included in the systematic review were conducted during the first trimester of pregnancy, with a vitamin D deficiency prevalence ranging from 22.7% to 59% [25,26,27,28]. Studies conducted in northern European countries or the US also reported heterogeneous rates of vitamin D deficiency, such as 10% in US in women in early pregnancy or 65% in pregnant women in Sweden (levels <50 nmol/L, which corresponds to ≤20 ng/mL). In Belgium, 47% of pregnant women in the first trimester were vitamin D deficient [29], in the Netherlands 8%–62% in the 12th week of pregnancy were deficient (deficiency defined as <25 nmol/L) [30], and in Norway 77.4% of pregnant women in the first trimester had a vitamin D deficiency [31]. Thus, our result lies within the range of vitamin D deficiency observed in early pregnancy. 

Concerning skin color, our results concur with previous data [30,32,33,34] showing that vitamin D deficiency varies by light and dark skin phototypes, i.e., dark skin color was significantly associated with vitamin D deficiency. Furthermore, studies consistently show that vitamin D levels among pregnant women in northern Europe and the US are lower in ethnic minority groups, which generally have darker skin. Dark-skinned individuals produce less 25(OH)D than individuals with light skin with the same sunlight exposure (UVB) [30,35,36,37]. 

Endogenous skin synthesis through UVB radiation and diet (or vitamin D supplement intake, respectively) are the two main sources of vitamin D. In our study, adjusting for season showed the best model fit, expressed as AIC (including also age as a covariate), which serves as a proxy for sunlight. Looking in more detail into vitamin D levels stratified by skin color and seasons, women in our study had higher vitamin D levels in summer compared to winter, and vitamin D levels were lower in women with dark than with light skin color, but differences between seasons were not statistically significant. Previous studies described lower vitamin D levels in winter for the general population [35], and for pregnant women most studies found higher vitamin D levels in summer than in winter, but not all tested for statistical significance [6,38,39,40]. 

In our study only a small percentage of women took vitamin D supplements and only half of the women ate fish at least once per week. As vitamin D supplement intake was a good AIC model fit, vitamin D levels stratified by intake and skin color were examined but differences were not statistically significant (Supplementary Figure S1). A recent meta-analysis and a systematic review observed that supplementing pregnant women with vitamin D leads to higher levels of vitamin D at term [41,42]. We hypothesize that in our study, it might be too early in pregnancy to see an effect. From a public health perspective, fortification of food could improve vitamin D levels in all pregnant women. To date, vitamin D fortification of food is more common in northern Europe (e.g., Norway, Denmark and Sweden) than in other countries of Europe, such as Switzerland [43].

We observed that age was an important covariate and that vitamin D levels were higher in older compared to younger pregnant women, although differences were not significant. Results of other studies looking at the relationship between age and vitamin D are contradictory and no association, non-linear association [44], or similar results as in our study [45] were observed. A possible explanation for an association with age may be that older individuals are more health conscious than younger pregnant women.

In our analysis, we used three different variables to categorize by skin type, namely the Fitzpatrick scale, melanin index and country of origin, and we observed that either variable was a predictor of vitamin D deficiency. However, the association of the (dichotomized) melanin index and vitamin D deficiency was strongly attenuated after adjusting for vitamin D supplement intake, BMI, and smoking status in addition to age and season. It might be that, despite the strong correlation between the melanin index and Fitzpatrick scale, the dichotomizing melanin index is less able to capture the extremes of skin color than the dichotomizing Fitzpatrick scale.

Women included in the study came from a great variety of countries of origin, which allowed different skin pigmentation colors to be represented. A further strength was the inclusion of a variety of confounders in our study, but due to collinearity, such as from country of origin, and the melanin index with light and dark skin color according to the Fitzpatrick scale, we could not include these variables in our final multivariable adjusted models. Nevertheless, we performed sensitivity analyses with these variables, which mostly confirmed our results with the Fitzpatrick scale. A further limitation was that women with dark skin (Fitzpatrick scale V) were limited in number and that other factors that affect vitamin D levels were not assessed, such as veiling of the women or physical activity. Hence, the observation of non-statistically significant differences in our population might be due to small numbers. Finally, we included only one hospital in our analysis, which limits the generalizability of our results. However, women attending our clinical pregnancy controls came from the general population. Also, residual confounding cannot be ruled out. 

## 5. Conclusions

The prevalence of vitamin D deficiency is common in women in early pregnancy. Almost two-thirds of all women in our study population had a vitamin D deficiency. To our knowledge, this is the first study that assessed prevalence rates for pregnant women in the Zurich area of Switzerland. We found a difference in 25(OH) vitamin D levels and prevalence depending on maternal skin type, emphasizing a consequent screening and supplementation program for pregnant women, in particular for women with a darker skin type.

## Figures and Tables

**Table 1:** General characteristics of pregnant women by vitamin D status.

Variables of Interest	Vitamin D Sufficiency ^1^	Vitamin D Deficiency ^2^	*p*-Value ^5^
*n* (%)	75 (37)	129 (63)	
25(OH)D ng/mL, geometric mean (95% CI)	26.1 (24.8–27.4)	10.5 (9.7–11.5)	<0.001
Light skin color ^3^, %	88	67	<0.05
Melanin levels, median (Q1, Q3)	32.9 (30.8, 37.2)	34.3 (30.8, 41.8)	0.07
Age, mean (SD)	31.1 (4.8)	29.4 (4.8)	<0.05
Week of pregnancy, median (Q1, Q3)	9 (8, 10)	9 (8, 10)	0.39
Parity, % nulliparous	55	52	0.32
Gravidity, % first pregnancy	43	40	0.11
BMI (kg/m^2^) before pregnancy, median (Q1, Q3)	20.7 (19.7, 23.1)	22.5 (20.4, 25.3)	<0.05
BMI (kg/m^2^) current, median (Q1, Q3)	21.5 (20.1, 23.9)	22.8 (20.7, 26.2)	<0.05
Country of origin, %			
Switzerland and Germany	35	14	
North America, North Europe, Caucasus, Central Asia and New Zealand (without Switzerland and Germany)	28	15	
South Europe, Australia, Latin America and the Caribbean	28	29	
South-, East Asia and Pacific	5	22	
Africa and Middle East	4	22	<0.001
Educational level achieved ^4^, %			
less than compulsory education	3	8	
low education	4	16	
middle education	35	33	
high education	59	44	<0.05
Educational level achieved of the partner ^4^, %			
less than compulsory education	4	8	
low education	3	14	
middle education	33	43	
high education	60	35	0.001
Smoking status, %			
Never smoker	47	67	
Ever smoker	45	22	
Current smoker	8	12	<0.05
Season			
Winter	24	25	
Spring	19	23	
Summer	20	19	
Fall	37	33	0.90
Days per week spent at least 1 h outdoor in the past half year, median (Q1, Q3)	2 (2, 5)	3 (2, 7)	0.44
Using sun protection in summer, %			
Never	13	31	
Sometimes	51	31	
Always	36	38	<0.05
Fish consumption at least once per week, %	51	45	0.41
Vitamin D supplement intake, %	9	9	0.97

^1^ 25(OH)D ≥ 20 ng/mL; ^2^ 25(OH)D < 20 ng/mL; ^3^ Light skin color defined as values I to III from the Fitzpatrick scale; ^4^ Low = compulsory education; middle = secondary education; high = tertiary education; ^5^ *t*-test was used for means, Mann-Whitney for medians. Chi^2^ was used for proportions or Fisher’s exact test, when one cell was <5.

**Table 2:** Associations between skin color and vitamin D deficiency in 204 pregnant women (reference: vitamin D level ≥ 20 ng/mL).

Dark Skin Color	OR	95% CI	AIC
age adjusted model	3.25	(1.46, 7.24)	259
age and season adjusted	3.29	(1.47, 7.36)	264
multivariable adjusted model ^1^	2.56	(1.08, 6.11)	266
multivariable adjusted model ^2^	2.60	(1.08, 6.22)	268

^1^ Adjusted for age, season, vitamin D supplement intake, BMI, smoking status; ^2^ Adjusted for age, season, vitamin D supplement intake, BMI, smoking status, parity.

## Supplementary Materials

The following are available online at http://www.mdpi.com/2072-6643/9/3/260/s1, Figure S1: Vitamin D levels by light and dark skin color and vitamin D supplementation status, Table S1: General characteristics of pregnant women with light and dark skin color, Table S2: Association between dichotomized country of origin and vitamin D deficiency in 204 pregnant women, Table S3: Association between dichotomized melanin index and vitamin D deficiency in 204 pregnant women.
