# Supplementary material for: Prevalence of Vitamin D Deficiency and Its Associations with Skin Color in Pregnant Women in the First Trimester in a Sample from Switzerland
Source: Nutrients. 2017 Mar 10;9(3):260. doi: 10.3390/nu9030260 (PMC5372923; doi:10.3390/nu9030260)
Supplement: Supplementary file 1 [file nutrients-09-00260-s001.docx]

Prevalence of Vitamin D Deficiency and Its Associations with Skin Color in Pregnant Women in the First Trimester in a Sample from Switzerland

**Aline Richard, Sabine Rohrmann and Katharina C. Quack Lötscher**

**Table S1.** General characteristics of pregnant women with light and dark skin color.

| **Variables of Interest** | **Light skin color ^1^** | **Dark skin color ^2^** | ***p*-Value ^3^** |
| --- | --- | --- | --- |
| n (%) | 152 (74.5) | 52 (25.5) |  |
|  |  |  |  |
| 25(OH)D, geometric mean (95% CI) | 16.26 (14.85-17.80) | 10.95 (9.25-12.97) | <0.001 |
| Melanin levels, median (Q1, Q3) | 32.21 (30.32,34.79) | 46.07 (39.82,53.54) | <0.001 |
| Age, mean (SD) | 30.6 (4.8) | 28.2 (4.6) | <0.05 |
| Week of pregnancy, median (Q1, Q3) | 9(8,10) | 9(9,10) | 0.11 |
| Parity, median (Q1, Q3) | 1 (1,2) | 1.5 (1,2) | 0.38 |
| Gravidity, median (Q1, Q3) | 2 (1,2.5) | 2 (1,3) | 0.52 |
| BMI before pregnancy, median (Q1, Q3) | 21.3 (19.8,23.5) | 24.2 (21.2,26.4) | <0.001 |
| BMI current, median (Q1, Q3) | 21.8 (20.3,23.8) | 25.0 (21.9,27.1) | <0.001 |
| Country of origin, % |  |  |  |
| Switzerland and Germany | 28.9 | 0 |  |
| North America and Nordeuropa and Caucasus and Central asia and New Zealand (without Switzerland and Germany) | 25.0 | 1.9 |  |
| South Europe and Australia Latin America and the Caribbean | 32.9 | 15.4 |  |
| South, East Asia and Pacific | 7.9 | 38.5 |  |
| Africa and middle east | 5.3 | 44.2 | <0.001 |
| Highest educational level achieved, % |  |  |  |
| < primary education | 2.6 | 15.4 |  |
| ≥ primary education | 6.6 | 25.0 |  |
| > primary to <= secondary education | 34.4 | 30.8 |  |
| > secondary to tertiary education | 56.3 | 28.8 | <0.001 |
| Highest educational level achieved of the partner, % |  |  |  |
| < primary education | 4.0 | 13.5 |  |
| ≥ primary education | 5.4 | 23.1 |  |
| > primary to <= secondary education | 39.6 | 38.5 |  |
| > secondary to tertiary education | 51.0 | 25.0 | <0.001 |
| Smoking status |  |  |  |
| never smoker, % | 51.3 | 82.7 |  |
| ever smoker, % | 36.2 | 13.5 |  |
| current smoker, % | 12.5 | 3.8 | <0.001 |
| Season |  |  |  |
| Winter | 27.0 | 17.3 |  |
| Spring | 19.7 | 25.0 |  |
| Summer | 19.1 | 21.2 |  |
| Fall | 34.2 | 36.5 | 0.57 |
| Days per week spent at least 1 hour outdoor in the past half year, median (Q1, Q3) | 2 (2,7) | 3 (2,7) | 0.08 |
| Using sun protection in summer, % |  |  |  |
| never | 15.0 | 52.9 |  |
| sometimes | 45.8 | 15.7 |  |
| always | 39.2 | 31.4 | <0.001 |
| Fish consumption at least once per week, % | 51.7 | 36.5 | 0.06 |
| Vitamin D supplement intake, % | 9.0 | 7.7 | 1.00 |

^1^ Fitzpatrick scale 1 to 3; ^2^ Fitzpatrick scale 4 and 5; ^3^ t-test was used for means, Mann-Whitney for medians. Chi^2^ was used for proportions or Fisher's exact test, when one cell was <5.

**Table S2.** Associations between dichotomized country of origin and vitamin D deficiency in 204 pregnant women.

| **Regression models** | **No insufficiency (Reference ≥ 20 ng/mL)**  **OR** | **OR** | **95% CI** | **AIC** |
| --- | --- | --- | --- | --- |
| Dark skin color |  |  |  |  |
| age adjusted model | 1 | 4.01 | (2.09,7.71) | 250 |
| age and season adjusted | 1 | 4.07 | (2.11,7.85) | 255 |
| multivariable adjusted model ^1^ | 1 | 3.50 | (1.75,7.02) | 257 |
| multivariable adjusted model ^2^ | 1 | 3.59 | (1.78,7.25) | 259 |

**^1^** Adjusted for age, season, vitamin d supplement intake, BMI, smoking status; ^2^ Adjusted for age, season, vitamin d supplement intake, BMI, smoking status, parity.

**Table** S**3.** Associations between dichotomized melanin index and vitamin D deficiency in 204 pregnant women.

| **Regression Models** | **No insufficiency (Reference ≥ 20 ng/mL)**  **OR** | **OR** | **95% CI** | **AIC** |
| --- | --- | --- | --- | --- |
| Dark skin color |  |  |  |  |
| age adjusted model | 1 | 1.91 | (1.06,3.45) | 273 |
| age and season adjusted | 1 | 2.00 | (1.09,3.65) | 268 |
| multivariable adjusted model ^1^ | 1 | 1.62 | (0.85,3.08) | 268 |
| multivariable adjusted model ^2^ | 1 | 1.61 | (0.85,3.09) | 270 |

^1^ Adjusted for age, season, vitamin D supplement intake, BMI, smoking status; ^2^ Adjusted for age, season, vitamin D supplement intake, BMI, smoking status, parity.

**
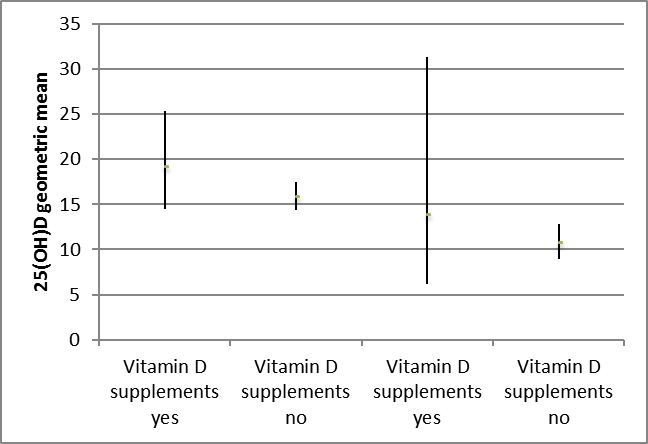
**

Light skin color

Dark skin color

**Figure S1.** Vitamin D levels by light and dark skin color and vitamin D supplementation status.
